# Predicting the Epidemiological Outbreak of the Coronavirus Disease 2019 (COVID-19) in Saudi Arabia

**DOI:** 10.3390/ijerph17124568

**Published:** 2020-06-25

**Authors:** Dabiah Alboaneen, Bernardi Pranggono, Dhahi Alshammari, Nourah Alqahtani, Raja Alyaffer

**Affiliations:** 1 Computer Science Department, College of Science and Humanities in Jubail, Imam Abdulrahman Bin Faisal University, Jubail P.O. Box 31961, Saudi Arabia; nfqahtani@iau.edu.sa (N.A.); rhalyafer@iau.edu.sa (R.A.); 2 Department of Engineering and Mathematics, Sheffield Hallam University, Sheffield S1 1WB, UK; b.pranggono@shu.ac.uk; 3 Computer Science and Information Department, College of Computer Science and Engineering, University of Ha’il, Hail 8145, Saudi Arabia; d.alshammri@uoh.edu.sa

**Keywords:** 2019 novel coronavirus, COVID-19, Saudi Arabia, logistic growth model, SIR model

## Abstract

The coronavirus diseases 2019 (COVID-19) outbreak continues to spread rapidly across the world and has been declared as pandemic by World Health Organization (WHO). Saudi Arabia was among the countries that was affected by the deadly and contagious virus. Using a real-time data from 2 March 2020 to 15 May 2020 collected from Saudi Ministry of Health, we aimed to give a local prediction of the epidemic in Saudi Arabia. We used two models: the Logistic Growth and the Susceptible-Infected-Recovered for real-time forecasting the confirmed cases of COVID-19 across Saudi Arabia. Our models predicted that the epidemics of COVID-19 will have total cases of 69,000 to 79,000 cases. The simulations also predicted that the outbreak will entering the final-phase by end of June 2020.

## 1. Introduction

Coronaviruses are a large family of viruses that are distributed among humans and animals such as livestock, birds, bats and other wild animals. These viruses cause serious illnesses for human when infecting respiratory, hepatic, gastrointestinal and neurological [1,2,3]. In December 2019, China notified the World Health Organization (WHO) about a novel coronavirus—severe acute respiratory syndrome coronavirus 2 (SARS-CoV-2)—that caused many cases of respiratory illnesses that were mostly related to people who had visited a live animal seafood market in Wuhan city [4]. The disease, now formally called COVID-19 (coronavirus disease 2019), caused an outbreak of a typical pneumonia through human-to-human transmission starting from Wuhan, a highly populated city with more than eleven million residents, then rapidly spread in China [5]. The Chinese authority put Wuhan city and several cities in Hubei province on lockdown, and all the public transportation was stopped to prevent any further spread of the virus [6,7]. However, the confirmed cases have increased daily in China and in many countries around the globe. On 11 March 2020, WHO officially declared COVID-19 outbreak as a global pandemic as the virus has spread to well over 200 countries around the world [8]. To date, COVID-19 has infected more than 4.5 million people and killed over 300,000 people across the world [8].

**Figure 1:**
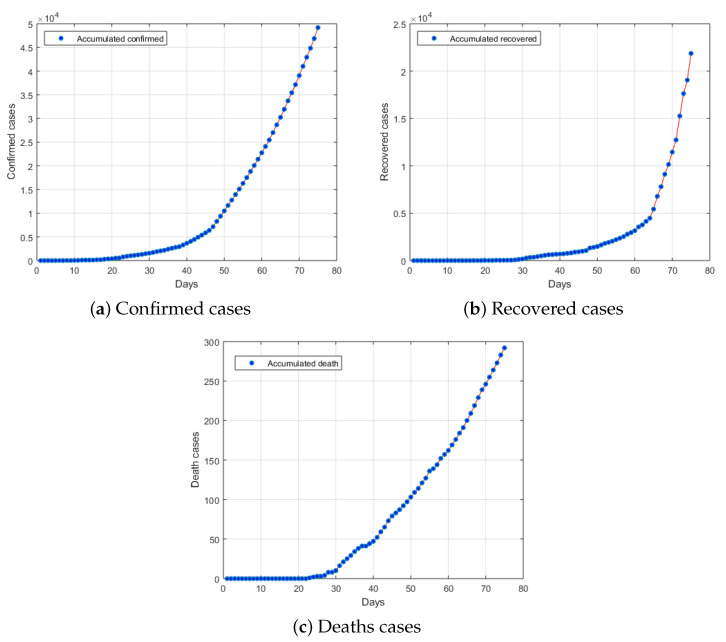
Number of (**a**) confirmed, (**b**) recovered and (**c**) deaths cases related to COVID-19 in Saudi Arabia from 2 March 2020 to 15 May 2020.

Saudi Arabia was among the countries that was affected by the virus. On 2 March 2020, the Saudi Ministry of Health reported the first confirmed COVID-19 case in the country. From the beginning of March, the number of confirmed COVID-19 cases gradually increased and the highest number of cases reported in one day was 2307 on 15 May 2020 with a total of 49,176 confirmed cases of COVID-19 infections and 292 deaths have been confirmed in Saudi Arabia (see Figure 1).

Mathematical models and simulations are considered an important tools to predict the possibility and severity of disease outbreak and provide main information for determining the type and intensity of disease intervention. Resulting in decreasing the transmission of the diseases and a more accurate approaches to manage the epidemic. Recently, mathematical modeling has been used to predict epidemics such as Foot-and-Mouth Disease (FMD), SARS, Zika and Ebola [9,10,11,12].

This article aims to give a local prediction of the epidemic peak for COVID-19 in Saudi Arabia by using the real-time data from 2 March 2020 to 15 May 2020.

The remainder of this article is arranged as follows. Section 2 presents the related work on COVID-19. Section 3 describes data and the models used in prediction of the epidemic peak for COVID-19 in Saudi Arabia. Section 4 presents our simulation results. Finally, Section 5 draws the conclusion.

## 2. Related Work

In [13], a model called FPASSA-ANFIS was proposed to predict the number of the confirmed COVID-19 cases for the upcoming 10 days after previous cases until 8 February 2020 in China in order to take the right course of action. The main idea of the model was to enhance the performance of Adaptive Neuro-Fuzzy Inference System (ANFIS) using parameters from the output of FPASSA, namely, Flower Pollination Algorithm (FPA) and Salp Swarm Algorithm (SSA). The result of the model was outstanding with regards to Mean Absolute Percentage Error, Root Mean Squared Relative Error, Root Mean Squared Relative Error, and coefficient of determination $\left(R^{2}\right)$. Moreover, the proposed model was evaluated with two other data sets, and the result showed good performance.

In [14], the end and the infection turning point of the COVID-19 epidemic in China and Hubei Province were predicted. A proper model was used and parameterized with the latest data of the daily and total infections in Hubei and China from NHC. The model predicted the end of the epidemic to be after March 10 with 51,600 infections, while the daily case turning points were predicted to be between 1 and 6 February 2020.

Roosa et al. in [15] generated a real-time prediction of cumulative confirmed COVID-19 cases in Hubei and China in general for the few upcoming days after 5 and 9 February 2020. They used three phenomenological models that were previously utilized to predict the epidemics of several other diseases. These models were the Generalized Logistic Growth model, Richards model, and sub-epidemic wave model. The models predicted that the average number of the additional cases would be from 7409 to 7496 in Hubei and 1128 to 1929 in China. Moreover, they concluded that by the end of 24 February, the predicted cases would be from 37,415 to 38,028 in Hubei and 11,588 to 13,499 in China.

In [16], the Case Fatality Rate (CFR) for COVID-19 in China was measured. They collected the confirmed and death cases from 10 January to 3 February then applied simple statistics technique such as linear regression to find the estimation. They found that the CFR of novel COVID-19 is lower than those of the previous SARS-CoV and Middle East Respiratory Syndrome coronavirus (MERSCoV).

Batista in [17] predicted the final infection numbers of COVID-19 in China. The Logistic Growth model and classic Susceptible-Infected-Recovered (SIR) dynamic model were used with data from Worldmeters website. The model predicted that the final estimation of coronavirus epidemic will be approximately 83,700 cases.

In [18], a mathematical model was developed to predict the effects of implementing government restrictions to contain COVID-19 epidemic on the number of infection cases in China. The model showed that the number of infection cases decreased if high restrictions are taken earlier instead of later.

When the novel COVID-19 started to spread in China in the first half of January 2020, many cases went unreported. In [19], a model was generated to estimate the real number of unreported cases with the help of existing information from the Serial Intervals (SI) of infection caused by the two previous coronaviruses (SARS and MERS). The model results showed that the unreported cases from 1 to 15 January 2020 were approximately 469 cases. In addition, they found that the cases increased by 21-fold after 17 January 2020. Moreover, in [20], they proposed a statistical model to estimate the rate of COVID-19 cases in China. In this model, they used the data of the evacuated Japanese citizens from Wuhan from 29 to 31 January 2020. The model estimated the infection rate to be 9.5, and the death rate to be from 0.3 to 0.6. The Japanese citizens totaled 565, and this number is insufficient to estimate an accurate rate.

In [21], the risk of transmission of COVID-19 was estimated. They proposed a model based on clinical information of the disease and confirmed cases of individuals. The estimated result of the reproduction number was higher than 6.47. In addition, the model predicted the confirmed cases in seven days (23 to 29 January 2020).

Thompson in [22] developed a mathematical model to estimate the sustained human-to-human transmission. The data of 47 patients were used, and the estimated result showed that the transmission rate is 0.4. Moreover, the transmission is only 0.012 in case the symptoms have not yet manifested in half of the tested data.

In [23], they proposed a model to estimate the COVID-19 death risk based on the data of 20 cases reported by 24 January 2020. Two different scenarios were estimated, and the results are 5.1 and 8.4. Moreover, the estimation of the reproduction for both scenarios are 2.1 and 3.2. The results indicated that the COVID-19 epidemic could become a pandemic.

## 3. Methodology

### 3.1. Data

We collected the daily number of confirmed, recovered, and deaths COVID-19 cases released by Saudi Ministry of Health’s Twitter account from 2 March 2020 to 15 May 2020 to construct a real-time database. The data were organized in a matrix with the rows representing the date and columns representing the number of the new confirmed cases, the number of accumulated cases, the number of accumulated recovered cases and the number of accumulated deaths cases as shown in Table 1 and Table 2.

### 3.2. Models

We generated short-term forecasts in real-time using two models namely, the Logistic Growth and SIR models.

#### 3.2.1. Logistic Growth Model

The phenomenological Logistic Growth model has been widely used to model population growth with limited resources and space. The model was originally developed by Haberman in [24] and have been used to predict 2015 Ebola epidemic [11,12]. The dynamics of the epidemic, typically expressed as a cumulative number of cases, can use the similar model when the primary method of control is quarantine—as in the case of a novel viral infection such as COVID-19.

In the Logistic Growth model, the epidemic can be defined by the differential equation

(1) $$\begin{array}{c} \frac{dC}{dt}=rC\left(1-\frac{C}{K}\right), \end{array}$$

where *C* is the cumulative cases at time *t*, *r* is the early growth rate, and *K* is the final epidemic size.

#### 3.2.2. Susceptible-Infected-Recovered Model

For comparison, we also simulate the well-known SIR model to represent the spreading process of COVID-19 epidemic in Saudi Arabia. SIR model framework is an epidemic spreading model inspired by the seminal work of Kermack and McKendric [25]. In epidemiology, SIR model is belong to compartmental epidemic models. The basic in compartmental epidemic model is proposed by Hamer in 1906 where he suggested that the infection spread should depend on the number of susceptible population and the number of infected population [26].

We estimate the parameters of model to get a best fit on reported data of COVID-19 outbreak in Saudi Arabia. In SIR model, we assume that the modeling timescale is short, no vital dynamics (births and deaths), and the host population size (*N*) is constant. In SIR-type models, individuals are classified into three separate groups (or compartments) based on their infectious status:

- Susceptible (*S*): group of individuals that not currently infected but may catch the disease.
- Infected (*I*): group of individuals that are currently infectious.
- Recovered or Removed (*R*): group of individuals that are no longer infectious. They are either recovered, become immune, or have died.

**Figure 2:**
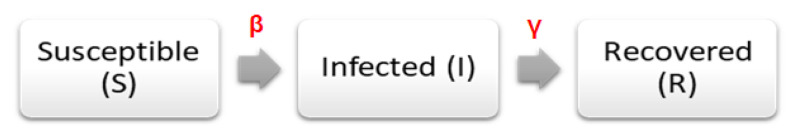
SIR model.

The SIR model is represented by the following system of nonlinear Ordinary Differential Equations (ODEs) [27]:

(2) $$\begin{array}{c} \frac{dS}{dt}=-\frac{\beta SI}{N} \end{array}$$

(3) $$\begin{array}{c} \frac{dI}{dt}=\frac{\beta SI}{N}-\gamma I \end{array}$$

(4) $$\begin{array}{c} \frac{dR}{dt}=\gamma I \end{array}$$

where *t* is time (in day), β is the contact rate, gamma is the remove rate or the inverse of infectious period, S(t) is the number of susceptible population at time *t*, I(t) is the number of infected population at time *t*, and R(t) is the number of recovered population at time *t* (see Figure 2).

In the SIR model, one key parameter to understand the basic epidemiological characteristics of the epidemic is the basic reproduction number ($R_{0}$). $R_{0}$ is the average number of secondary persons in a complete susceptible population infected by a single infected person during its spreading life. It indicates how contagious is the infectious diseases. The true value of $R_{0}$ is uncertain until the outbreak is over. $R_{0}$ depends on three factors:

- Duration of infectiousness;
- Probability of infection being transmitted during contact between an infected person and a susceptible person;
- The average rate of contact between infected and susceptible individuals.

$R_{0}$ is represented as

(5) $$\begin{array}{c} R_{0}=\frac{\beta }{\gamma } \end{array}$$

when $R_{0}$ > 1 virus is currently spreading in the population and when $R_{0}$ < 1 virus is stop spreading due to run out of susceptible and the decrease of new cases.

## 4. Results

### 4.1. Results of Logistic Growth Model

We used MATLAB to simulate the Logistic Growth and SIR model based on [17,28] models. In the epidemic simulation graphs, regions color separate epidemic into three phases:

- Red: fast growth phase;
- Yellow: transition to steady-state phase;
- Green: ending phase (plateau stage).

**Figure 3:**
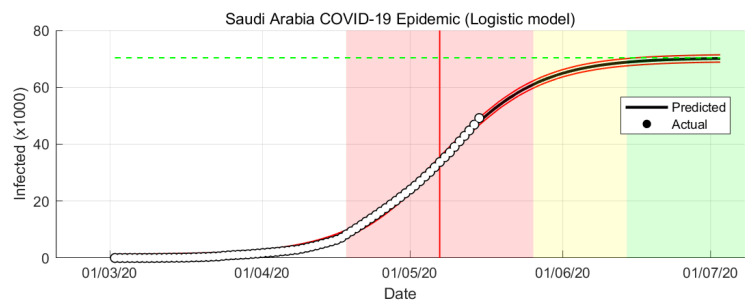
COVID-19 epidemic in Saudi Arabia prediction based on Logistic Growth model.

**Figure 4:**
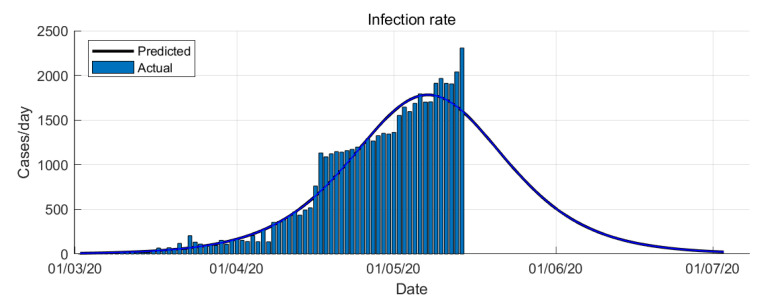
COVID-19 epidemic in Saudi Arabia prediction based on Logistic Growth model (infection rate).

The Logistic Growth model results of COVID-19 epidemic in Saudi Arabia are shown in Figure 3 and Figure 4.

The simulation is data-driven which is rely on the historical time-series data. From the simulations, it is shown that the Logistic Growth prediction is more optimistic compare to the other models. From the Logistic Growth model prediction, the peak of the infection rate is 7 May 2020 (see Figure 3). The transition to steady-state phase starts on 28 May 2020 and the ending phase starts on 14 June 2020. The Logistic Growth model predicts that final number of case is around 69,000 cases.

### 4.2. Results of Susceptible-Infected-Recovered Model

**Figure 5:**
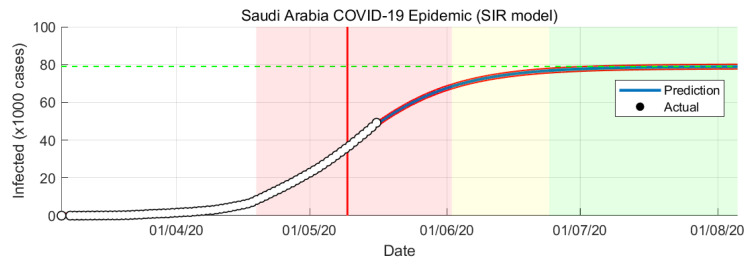
COVID-19 epidemic in Saudi Arabia prediction based on SIR model.

**Figure 6:**
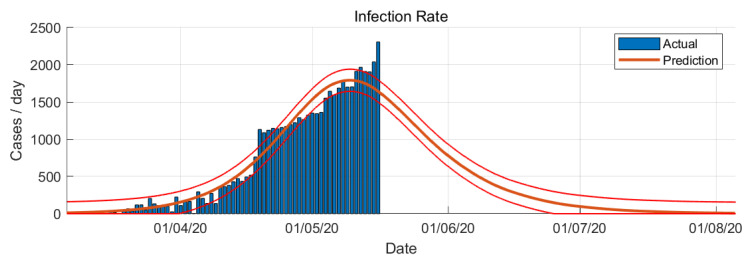
COVID-19 epidemic in Saudi Arabia prediction based on SIR model (infection rate).

Several assumptions were taken in the simulation of SIR model: constant total population, uniform mixing of the people, and equally likely recovery of infected. The SIR model of COVID-19 epidemic simulation in Saudi Arabia are shown in Figure 5 and Figure 6.

In SIR-model the parameters β and γ are estimated from the actual number of confirmed cases. The variable $R_{0}$ changes with respect to time. It will change every day based on the number of cases confirmed. From the SIR-model prediction, the peak of the infection rate is 1 May 2020. The transition to steady-state phase starts on 2 June 2020 and the ending phase starts on 24 June 2020. The SIR-model predict that final number of case is around 79,000 cases.

## 5. Conclusions

Predicting the epidemic evolution based on limited data and with no past epidemiological data is not trivial task. We present predictions for reported cases of COVID-19 in Saudi Arabia from 2 March to 15 May 2020 using mathematical modeling and simulation. We used two models: Logistic Growth and SIR models. Across both predictions, Logistic Growth and SIR-model provide different results (Figure 3 and Figure 5). Both models are similar in predicting the epidemic trends but with a slightly different timing, the SIR model predicts about a few days late than the Logistic Growth. Both models also have a gap in predicting the final number of cases with SIR model has a higher number of cases compared to the Logistic Growth.

In conclusion, while our models predict the COVID-19 outbreak in Saudi Arabia still in fast growth phase, our predictions need to be interpreted with caution given the dynamic case definition and reporting patterns. Without mass testing, the confirmed case number might be only a subset of the true total infected cases. Also, the asymptomatic infected individuals who are not tested and then recovered do not get counted. Under-reporting and asymptomatic people are observed in many countries worldwide and may lead to under-estimation of the accumulated cases. Therefore, mass testing is needed to identify patients and to contain the spread of the disease. Our prediction based on current data suggests that the epidemic continue to spreading in Saudi Arabia. It is suggested that warmer weather may contribute to slowing down the spread of coronavirus but this would need further investigation when more data is available. It should be noted that the MERS coronavirus has spread in Saudi Arabia in the summer (August) [29].

## Figures and Tables

**Table 1:** Number of cases in Saudi Arabia from 2 March 2020 to 15 April 2020.

Date	New	Accumulated Confirmed	Accumulated Recovered	Accumulated Deaths
2/3/2020	1	1	1	0
4/3/2020	1	2	0	0
5/3/2020	3	5	0	0
6/3/2020	2	7	0	0
7/3/2020	4	11	0	0
8/3/2020	4	15	0	0
9/3/2020	5	20	0	0
10/3/2020	1	21	1	0
11/3/2020	24	45	0	0
12/3/2020	17	62	0	0
13/3/2020	24	86	1	0
14/3/2020	17	103	1	0
15/3/2020	15	118	2	0
16/3/2020	15	133	2	0
17/3/2020	38	171	0	0
18/3/2020	67	238	6	0
19/3/2020	36	274	8	0
20/3/2020	70	344	8	0
21/3/2020	48	392	16	0
22/3/2020	119	511	17	0
23/3/2020	51	562	19	0	
24/3/2020	205	767	28	1
25/3/2020	133	900	29	2
26/3/2020	112	1012	33	3
27/3/2020	92	1104	35	3
28/3/2020	99	1203	37	4
29/3/2020	96	1299	66	8
30/3/2020	154	1453	115	8
31/3/2020	110	1563	165	10
1/4/2020	157	1720	264	16
2/4/2020	165	1885	328	21
3/4/2020	154	2039	351	25
4/4/2020	140	2179	420	29
5/4/2020	206	2385	488	34
6/4/2020	138	2523	551	38
7/4/2020	272	2795	615	41
8/4/2020	137	2932	631	41
9/4/2020	355	3287	666	44
10/4/2020	364	3651	685	47
11/4/2020	382	4033	720	52
12/4/2020	429	4462	761	59
13/4/2020	472	4934	805	65
14/4/2020	435	5369	889	73
15/4/2020	493	5862	931	79

**Table 2:** Number of cases in Saudi Arabia from 16 April 2020 to 15 May 2020.

Date	New	Accumulated Confirmed	Accumulated Recovered	Accumulated Deaths
16/4/2020	518	6380	990	83
17/4/2020	762	7142	1049	87
18/4/2020	1132	8274	1329	92
19/4/2020	1088	9362	1398	97
20/4/2020	1122	10,484	1490	103
21/4/2020	1147	11,631	1640	109
22/4/2020	1141	12,772	1812	114
23/4/2020	1158	13,930	1925	121
24/4/2020	1172	15,102	2049	127
25/4/2020	1197	16,299	2215	136
26/4/2020	1223	17,522	2357	139
27/4/2020	1289	18,811	2531	144
28/4/2020	1266	20,077	2784	152
29/4/2020	1325	21,402	2953	157
30/4/2020	1351	22,753	3163	162
1/5/2020	1344	24,097	3555	169
2/5/2020	1362	25,459	3765	176
3/5/2020	1552	27,011	4134	184
4/5/2020	1645	28,656	4476	191
5/5/2020	1595	30,251	5431	200
6/5/2020	1687	31,938	6783	209
7/5/2020	1793	33,731	7798	219
8/5/2020	1701	35,432	9120	229
9/5/2020	1704	37,136	10,144	239
10/5/2020	1912	39,048	11,457	246
11/5/2020	1966	41,014	12,737	255
12/5/2020	1911	42,925	15,257	264
13/5/2020	1905	44,830	17,622	273
14/5/2020	2039	46,869	19,051	283
15/5/2020	2307	49,176	21,869	292
